# Synergistic Effect of 3′,4′-Dihidroxifenilglicol and Hydroxytyrosol on Oxidative and Nitrosative Stress and Some Cardiovascular Biomarkers in an Experimental Model of Type 1 Diabetes Mellitus

**DOI:** 10.3390/antiox10121983

**Published:** 2021-12-13

**Authors:** José Pedro De La Cruz Cortés, Leticia Vallejo-Carmona, María Monsalud Arrebola, Esther Martín-Aurioles, María Dolores Rodriguez-Pérez, Laura Ortega-Hombrados, Cristina Verdugo, María África Fernández-Prior, Alejandra Bermúdez-Oria, José Antonio González-Correa

**Affiliations:** 1 Departmento de Farmacología, Instituto de Investigación Biomédica (IBIMA), Facultad de Medicina, Universidad de Málaga, 29010 Málaga, Spain; jpcruz@uma.es (J.P.D.L.C.C.); lauraortegah@outlook.es (L.O.-H.); cristinaverdugocabello@uma.es (C.V.); correa@uma.es (J.A.G.-C.); 2 Facultad de Enfermería, Universidad Ana G. Mendez, Recinto Cupei, San Juan PR 00928, Puerto Rico; vallejo1@uagm.edu; 3 UGC Laboratorio Clínico, Hospital de la Axarquía, AGSEMA, 29740 Málaga, Spain; mariam.arrebola.sspa@juntadeandalucia.es; 4 Distrito Sanitario Málaga-Guadalhorce, UGC La Roca, 29009 Málaga, Spain; estherd.martin.sspa@juntadeandalucia.es; 5 Consejo Superior de Investigaciones Científicas (CSIC), Instituto de la Grasa, 41013 Seville, Spain; mafprior@ig.csic.es (M.Á.F.-P.); aleberori@ig.csic.es (A.B.-O.)

**Keywords:** hydroxytyrosol, extra-virgin olive oil polyphenols, 3′,4′-dihidroxifenilglicol, oxidative stress, cardiovascular

## Abstract

The objective of this study was to assess a possible synergistic effect of two extra-virgin olive oil polyphenols, 3,4,-dyhydroxyphenylglycol (DHPG) and hydroxytyrosol (HT), in an experimental model of type 1 diabetes. Seven groups of animals were studied: (1) Nondiabetic rats (NDR), (2) 2-month-old diabetic rats (DR), (3) DR treated with 5 mg/kg/day p.o. HT, (4) DR treated with 0.5 mg/kg/day p.o. DHPG, (5) DR treated with 1 mg/kg/day p.o. DHPG, (6) DR treated with HT + DHPG (0.5), (7) DR treated with HT + DHPG (1). Oxidative stress variables (lipid peroxidation, glutathione, total antioxidant activity, 8-isoprostanes, 8-hydroxy-2-deoxyguanosine, and oxidized LDL), nitrosative stress (3-nitrotyrosine), and some cardiovascular biomarkers (platelet aggregation, thromboxane B_2_, prostacyclin, myeloperoxidase, and vascular cell adhesion protein 1 (VCAM-1)) were analyzed. The diabetic animals showed an imbalance in all of the analyzed variables. HT exerted an antioxidant and downregulatory effect on prothrombotic biomarkers while reducing the fall of prostacyclin. DHPG presented a similar, but quantitatively lower, profile. HT plus DHPG showed a synergistic effect in the reduction of oxidative and nitrosative stress, platelet aggregation, production of prostacyclin, myeloperoxidase, and VCAM-1. This synergism could be important for the development of functional oils enriched in these two polyphenols in the proportion used in this study.

## 1. Introduction

The effect of high adherence to a Mediterranean-type diet (high content in fruits, vegetables, legumes, fish, and extra-virgin olive oil) has been widely demonstrated to reduce the incidence of any type of cardiovascular disease and mortality from any cause [1,2]. The main source of fat in this diet is extra-virgin olive oil (EVOO). There is much evidence that shows that a large part of the beneficial effects of the Mediterranean diet are due to the intake of EVOO [3]. It is even established that the necessary amount of EVOO to demonstrate these effects, especially at the cardiovascular level, is 25–50 g/day [4,5].

Many studies have been published, both experimental studies and studies of humans, on the influence of EVOO on certain biomarkers that are altered in cardiovascular diseases: oxidation of low-density lipoproteins (oxLDL), platelet and endothelial dysfunction, oxidative stress, inflammatory mediators, etc. [4,6,7].

Among all of the chemical components of EVOO, the polyphenolic compounds are of the highest importance to explaining the benefits of EVOO [8]. The most abundant polyphenol in its free form is hydroxytyrosol (HT), of which its antioxidant, anti-inflammatory, antiplatelet and neuroprotective effects, among others, have been amply demonstrated [8,9]. In some experimental studies, it has been shown that certain beneficial effects of HT do not fully explain the complete effect of EVOO, using doses of HT such as those contained in EVOO [10], postulating a possible positive interaction between the polyphenols contained in EVOO.

It has recently been shown that, in an in vitro model of hypoxia-reoxygenation in rat brain slices, the incubation of the main polyphenols of EVOO, in the same proportion in which they are found in EVOO, produce a higher neuroprotective and antioxidant effect than isolated incubation of HT [10]. One of the compounds that most favors the neuroprotective and antioxidant effect of HT is 3’,4’-dihydroxyphenylglycol (DHPG), which has a chemical structure very similar to that of HT and has a demonstrated antioxidant and inhibitory effect on platelet aggregation [11,12,13]. This positive interaction has been demonstrated in other effects, such as the inhibition of platelet aggregation, the production of inflammatory mediators, or lipid peroxidation [10,14].

One of the main risk factors for cardiovascular disease is diabetes mellitus. Therefore, the objective of the present study was to assess the possible synergistic effect of HT and DHPG, in a similar proportion to that found in EVOO, on oxidative and nitrosative stress and on some cardiovascular biomarkers in an experimental model of type 1 diabetes mellitus.

## 2. Material and Methods

### 2.1. Study Design

The animals were 2-month-old adult male Wistar rats (body weight 200–250 g). All rats were used in accordance with current Spanish legislation for animal care, use, and housing (EDL 2013/80847. BOE-A-2013-6271). The recommendations in the Guide for the Care and Use of Laboratory Animals (NIH publication No. 86–23, revised 1985) were followed, as well the Spanish Law on the Protection of Animals, where applicable. The study protocol was approved by the University of Malaga Ethics Committee for the Use of Animals (Ref. CEUMA31-2018-A) and the Consejería de Agricultura, Ganadería, Pesca y Desarrollo Sostenible, Junta de Andalucía [Department of Agriculture, Livestock, Fisheries and Sustainable Development of the Regional Government of Andalusia] (Ref. 9/07/2019/124).

Animals were distributed in seven groups (10 rats/group): (1) saline-treated normoglycemic control rats (NDR), (2) saline-treated control diabetic rats (DR), (3) DR treated with 5 mg/kg/day p.o. HT, (4) DR treated with 0.5 mg/kg/day p.o. DHPG, (5) DR treated with 1 mg/kg/day p.o. DHPG, (6) DR treated with 5 mg/kg/day p.o. HT plus 0.5 mg/kg/day p.o. DHPG and (7) DR treated with 5 mg/kg/day p.o. HT plus 1 mg/kg/day p.o. DHPG. These doses of each compound were chosen according to the range of concentrations described in EVOO with different polyphenol content [15,16]: from 1/10 to 1/5 (DHPG/HT) depending on the type of olive from where the EVOO was extracted and to the effects demonstrated in some ex vivo experiments in healthy and diabetic rats [17,18]. HT and DHPG were administered in the drinking water once daily for seven days before diabetes was induced and continued daily until the end of the diabetic period (2 months).

Experimental diabetes was induced with a single intravenous injection of streptozotocin (50 mg/kg). Blood glucose concentration was measured by placing a FreeStyle^®^ glucometer (Abbot Laboratories S.A., Madrid, Spain) in contact with blood from the saphenous vein. Animals were considered to have diabetes if the blood glucose was higher than 200 mg/dL for two consecutive days. Rats in the nondiabetic control group received a single intravenous injection of an isotonic saline solution, and blood glucose was measured in the same way as in the diabetic animals.

In order to maintain blood glucose levels high enough to induce vascular damage and avoid high mortality due to blood glucose levels close to 500–600 mg/dL, diabetic animals were treated with 4 IU/day s.c. of a soluble long-acting basal insulin analogue (Levemir^®^, Novo Nordisk Spain, Madrid, Spain). No animal died before the end of the study period. Control animals received the same volume of an isotonic saline solution s.c.

At the end of the second month, all animals from each group were anesthetized with pentobarbital sodium (40 mg/kg i.p.). Two milliliters of blood were drawn from the vena cava and anticoagulated with sodium citrate. Then, a segment of the abdominal aorta 0.5 cm anterior to the bifurcation of the femoral arteries was obtained.

### 2.2. Material and Preparation of Polyphenols

11-dehydro Thromboxane B_2_, 3-nitrotyrosine and 6-keto-prostaglandin F_1α_ (6-keto-PGF_1α_) enzyme immunoassay kits were obtained from Cayman Chemical (Ann Arbor, MI, USA). 8-isoprostane enzyme immunoassay and Total Antioxidant Capacity colorimetric kits were obtained from Cell Biolabs, Inc. (Arjons Drive, San Diego, CA, USA). VCAM-1, Myeloperoxidase (MPOx), and oxidized low-density lipoprotein (oxLDL) enzyme immunoassay kits were obtained from Abyntek Biopharma, S.L. (Vizcaya, Bilbao, Spain). Glutathione concentration and glutathione peroxidase activity kits and 8-hydroxy 2 deoxyguanosine enzyme immunoassay kits were obtained from Abcam plc (Cambridge, UK). Collagen was obtained from Menarini Diagnóstica (Barcelona, Spain). All other reagents were obtained from Sigma Chemical Corp. (St. Louis, MO, USA).

Hydroxytyrosol was isolated by hydrothermal treatment of the liquid phase obtained from *alperujo* (a by-product of the two-phase olive oil separation system) according to a described technique [10,19,20].

3′,4′-dihidroxifenilglicol was obtained from the olive oil by-product by a two-phase extraction system that is used in olive oil mills for the isolation of the DHPG. The method to purify DHPG has been described and patented by Fernández-Bolaños et al. (2010) (WO2010070168A1). This method is based on physical chromatographic systems that allow the extraction of natural compounds without any organic solvent or chemical or enzymatic reactions, obtaining a purity degree over 95% when referring to dry matter.

### 2.3. Analytical Techniques

All techniques were run in a single-blind manner, i.e., the persons who performed the assays were unaware of the origin and nature of the samples.

#### 2.3.1. Samples

The following samples were extracted from each animal:

− Whole blood. Blood was collected in tubes without anticoagulants and with coagulation activator gel. The samples were centrifuged at 3500× *g* for 10 min and the supernatant was separated and frozen in aliquots at −80 °C until the determination of the corresponding variables.
− Urine. Rats were individually placed in modular metabolic cages (Tecniplast S.p.A., Buguggiate, Italy) and 24-h urine was collected. Total diuresis was measured, and the samples were centrifuged at 3500× *g* for 10 min at 4 °C and frozen at −80 °C in aliquots until the corresponding analytical determinations were made.
− Aortic tissue. A segment of the aorta from the division of the renal arteries to 1 cm upwards was obtained from each animal.

#### 2.3.2. Serum Biochemistry

All biochemical parameters were analyzed using the Atellica^®^CH autoanalyzer from Siemens Healthineers (Erlangen, Germany). Triglycerides, total cholesterol, cholesterol HDL, and cholesterol LDL were measured according to the standard manufacturer protocols.

#### 2.3.3. Platelet Aggregometry

Platelet aggregation was carried out in anticoagulated whole blood samples with the electrical impedance method (Chrono-Log 540 aggregometer, Chrono-Log Corp., Haverton, PA, USA). Collagen (10 µg/mL) was used as the aggregation-inducing agent. The maximum intensity of platelet aggregation was determined as the maximum change in resistance (ohms) in the electrode.

#### 2.3.4. Urine Eicosanoid Concentration

Urinary 11-dehydro-thromboxane B_2_ and 6-keto-prostaglandin F_1α_ concentrations were measured as an index of the global production of thromboxane and prostacyclin. These determinations were made using a commercial enzyme immunoassay kit according to the manufacturer’s protocols.

#### 2.3.5. Oxidative and Nitrosative Stress

Malondialdehyde is the main product of reaction with thiobarbituric acid (TBARS) and was used as an index of serum lipid peroxide concentration. A colorimetric commercial kit was used, based on the reaction with thiobarbituric acid and spectrophotometric determination at 532 nm.

Urinary 8-isoprostane was determined as a global index of oxidative stress [21]; serum 8-hydroxy-2-deoxyguanosine concentrations were determined as an index of oxidative stress/DNA damage. Both determinations were carried out using commercial enzyme immunoassay kits according to the manufacturer’s protocols.

Serum glutathione concentration, glutathione peroxidase activity (GSHpx), and total antioxidant capacity (TAC) were determined as a global index of antioxidant defense. Glutathione determination was measured by the absorbance at 412 nm. GSHpx activity was measured using a colorimetric method at 340 nm in a kinetic mode. The TAC assay was based on the reduction of Cu^2+^ to Cu^+^ by antioxidants such as uric acid and the reaction with a chromogen, determining the absorbance at 490 nm.

Serum 3-nitrotyrosine concentrations were determined as an index of peroxynitrite formation, using a commercial enzyme immunoassay kit according to the manufacturer’s protocols.

#### 2.3.6. Other Cardiovascular Biomarkers

Serum oxidized low-density lipoprotein (oxLDL) was measured as an index of the oxidative status caused by free radicals. The concentration of myeloperoxidase (MPOx) was determined as an index of the activation of polymorphonuclear leukocytes in the presence of inflammatory mediators. The vascular adhesion molecule VCAM-1 reflected endothelial activation and was used as a biomarker of the chronic inflammatory process in the development of vascular disease. These biomarkers were quantified with the appropriate commercial enzyme immunoassay kits, according to the manufacturer’s protocols.

#### 2.3.7. Aortic Morphometric Analysis

The excised aortic tissue was fixed in 10% paraformaldehyde for 48 h and was processed with the classical paraffin-embedding method. Sections were cut at 7 μm and stained with hematoxylin and eosin. The sections were examined with a digital system. Histomorphometric analysis was carried out with the Visilog v. 6.3 computer program licensed to the Central Computer Service of the University of Malaga.

From each arterial sample, 10 randomly chosen sections from 5 to 7 slides (containing 5 to 8 sections per slide) were analyzed. In each section we quantified the variables (i) area of the lumen (A_L_) and (ii) area of the entire arterial section (A_W_). The area of the arterial wall (A_AW_) was calculated as follows:

A_AW_ = A_W_ − A_L_

The program adapted the area obtained in each case to a circle, calculating the radius (vessel wall thickness-VWT) as follows:

VWT^2^ = Area/3.1416

In addition, stained aortic sections were used to count the number of smooth muscle cell nuclei in the tunica media. The image was segmented into a new binary image, with black representing the nuclei. Before calculation, any artifacts or particles smaller than the predetermined size were eliminated. The number of cell nuclei was then calculated within four fields of approximately 10,000 µm^2^ at 0°, 90°, 180°, and 270° in each section.

### 2.4. Statistical Analysis

The data in the text, tables, and figures are expressed as the means ± standard deviations (SD) of 10 animals. All statistical analyses were performed with the Statistical Package for Social Sciences v. 25.0 (SPSS Co., Chicago, IL, USA). One-way analyses of variance followed by Bonferroni transformations and unpaired Student’s *t*-tests were used. In all cases, statistical significance was assumed at a value of *p* < 0.05.

## 3. Results

The zoometric variables analyzed (Table 1) were significantly modified in the untreated diabetic animals. Chow intake and water intake were higher than in nondiabetic animals and they showed the two fundamental signs of type 1 diabetes mellitus in humans: polydipsia (greater intake of drink) and polyphagia (greater intake of food). Body weight and food intake did not change significantly in any of the treatment groups, while water intake tended to decrease with respect to the diabetic control group but did not reach the values of the nondiabetic group.

Blood glucose (Table 2) increased significantly in the control diabetic animals, remaining elevated throughout the follow-up period (x4.8 compared to nondiabetic animals). The administration of HT, DHPG and their association did not significantly modify blood glucose levels.

Diabetic animals showed significant increase in triglyceride levels with respect to the nondiabetic group (Table 2). The administration of HT only produced a significant increase in serum HDL-cholesterol levels (63% increase compared to control diabetic animals). DHPG did not significantly modify any of the lipid profile variables. The HT-DHPG association (0.5 mg/kg/day) significantly reduced triglyceride levels compared to the diabetic control group and HT alone.

Regarding the oxidative and nitrosative stress variables (Table 3), diabetic animals showed a clear profile of greater oxidative and nitrosative stress compared to nondiabetic animals: higher levels of TBARS (x2.2), oxLDL (76.4%), 8-OHdG (76.2%), urinary 8-isoprostanes (41.5%), GSHpx (x2.6), and 3-nitrityrosine (x3.3), and a reduction in GSH (30.9% lower) and TAC (38.8% lower) levels. The administration of HT showed a clear antioxidant profile compared to the control diabetic animals: significant reductions in TBARS, oxLDL, 8-OHdG, 8-isoprostanes, and 3 nitrotyrosine and increased TAC and GSH. DHPG showed a behavior profile similar to HT, but quantitatively lower. The antioxidant effect of DHPG was increased in the presence of HT, resulting in the reduction of TBARS (DHPG 1 mg/kg/day), oxLDL, 8-isoprostanes (DHPG 1 mg/kg/day), 8-OHdG, and 3 nitrotyrosine and in the increase of GSH.

Regarding the cardiovascular variables (Table 4), the control diabetic animals showed alterations in all of the analyzed variables: increases in the maximum intensity of platelet aggregation (80.4%), 11-dH-TxB_2_ (x2.5), MPOx (x3.5), and VCAM-1 (x2.0) and reduced production of 6-keto-PG F_1α_ (51.4%). The administration of HT reduced the so-called prothrombotic variables (platelet aggregation, thromboxane B_2_, MPOx, and VCAM-1) and slowed the decrease in prostacyclin production. Administration of DHPG to diabetic animals significantly reduced platelet aggregation and VCAM-1 and decreased prostacyclin inhibition. The addition of DHPG increased the antiplatelet effect, the inhibitory effect on VCAM-1 levels (1 mg/kg/day), and the stimulatory effect on prostacyclin synthesis exerted by HT.

**Figure 1:**
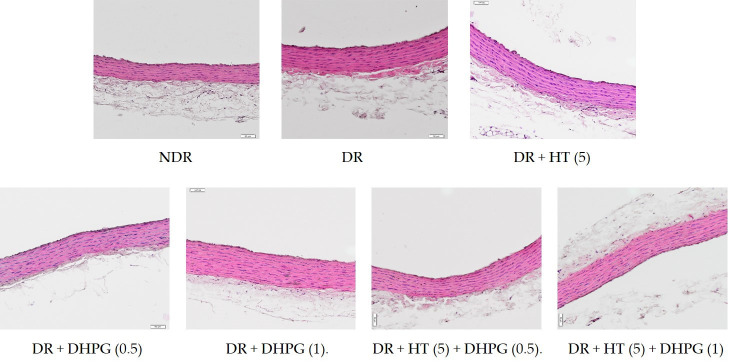
Representative histological images of aortic walls. Hematoxylin and eosin staining, 20×. Nondiabetic rats (NDR), diabetic control rats (DR), and DR treated with hydroxytyrosol (HT) 5 mg/kg/day p.o. (HT-5), 3′,4′-dihidroxifenilglicol (DHPG) 0.5 or 1 mg/kg/day p.o. (DHPG-0.5, DHPG-1), or their association.

The area of the middle layer of the arteries analyzed in the group of diabetic control animals (Table 5) was significantly higher when compared to the nondiabetic control animals (33.3% higher). Similarly, the arteries of diabetic animals presented a higher cell density in the middle layer than those of normoglycemic animals (31.2% higher). All of the treatments administered to diabetic animals significantly decreased both morphometric parameters, without statistically significant differences between them. Some examples of the arterial images of these groups of animals are shown in Figure 1.

## 4. Discussion

The studies that address the possible interactions of the biological effects of the main polyphenols in EVOO have two main objectives [22,23]. First, to provide the evidence to obtain functional oils that improve the healthy profile of EVOO. On the other hand, these studies attempt to explain the final effect of EVOO through an interaction between its main polyphenolic compounds because studies that analyzed these compounds separately showed biological effects (antioxidant, anti-inflammatory, on cardiovascular markers, neuroprotective, etc.) that were not as significant as those demonstrated by EVOO [10,20]. This second aspect is the most directly related to our study.

The main polyphenols implicated in the beneficial effects of EVOO were shown to be HT, DHPG, and oleocanthal [23]. Likewise, in brain tissue subjected to an in vitro hypoxia-reoxygenation model, DHPG was shown to significantly increase the neuroprotective effect of HT, possibly due to a synergistic effect at the level of its antioxidant action [10].

The results obtained in the present study showed that the experimental model of diabetes used in this study presented an evident oxidative and nitrosative stress, as well as an increase in the biomarkers involved in cardiovascular disease in humans, which justified its use to study the association of HT and DHPG in a disease that is a risk factor for cardiovascular disease.

Both HT [24,25] and DHPG [26,27] have separately demonstrated antioxidant, anti-inflammatory, and antithrombotic effects, all of them related to important mechanisms involved in the development of cardiovascular disease [28]. Furthermore, EVOO was shown to modify these pathophysiological mechanisms [4,5]. It has been postulated that a possible synergistic effect between some of the polyphenols that make up EVOO could improve the final cardiovascular effect of EVOO, making it possible to design a new EVOO with greater cardiovascular protective power [23]. Specifically, with HT and DHPG, this synergy was demonstrated at the level of platelet aggregation, both in vitro [12,14] and ex vivo [14], as well as in its antioxidant [10,13], neuroprotective [10] and anti-inflammatory [13] effects.

In the present study, these effects were combined in the same experimental model and this positive interaction was demonstrated in almost all biomarkers of cardiovascular disease: oxidative and nitrosative stress, adhesive proteins, and biomarkers of ischemic cardiovascular disease. Likewise, this synergistic effect was demonstrated with doses of HT/DHPG in the same ratios that are found in EVOO of different origins [19,20].

Regarding the antioxidant effect, both compounds showed an inhibition of oxidative variables and an increase in antioxidants, as well as an inhibition in the production of nitrosative stress. In all cases, fundamentally by associating DHPG 1 mg/kg/day, the profile shown of both polyphenols was improved. It was described that both HT and DHPG have an ability to capture free radicals [13], as well as to inhibit the production of lipid peroxidation [10], which would explain that there is a synergistic effect between them (Table 3). This study showed that this effect also occurred on glutathione and nitrosative stress levels. It is important to highlight that it was also observed in the serum concentration of oxLDL, a molecule of importance in the pathophysiology of vascular disease, whether in diabetic patients or not [29].

Related to its antioxidant power, both in isolation and in association, HT and DHPG inhibited platelet activity and increased vascular prostacyclin production (Table 4). The effect of HT on platelet aggregation was demonstrated both in vitro in human blood [30] and ex vivo in healthy and diabetic rats [20,31]. Likewise, DHPG showed an antiplatelet effect in human blood [12,14] that was potentiated in the presence of HT [12,14].

The present study demonstrated that this synergistic effect occurs under ex vivo conditions and in an experimental diabetes model. The mechanism by which this interaction was explained focuses on the expression of the proteins that regulate the platelet fibrinogen receptor [12]. The present study showed that this greater effect of the HT-DHPG association could be due, in part, to a greater production of vascular prostacyclin under ex vivo conditions, directly related to an antioxidant effect, since free radicals are one of the main causes of the degradation of vascular prostacyclin [32].

The synergistic effect on the other cardiovascular biomarkers of importance, such as MPOx and VCAM-1, in the development of atherosclerotic vasculopathy, is also important. All of these findings contribute a very important aspect to these polyphenolic compounds in cardiovascular disease, since they participate in several stages of the disease: oxidative and nitrosative stress, vascular inflammation, and pro and antithrombotic mechanisms. These effects were demonstrated for HT in the same experimental model of diabetes [20]. In this study we demonstrated that DHPG also showed these effects in ex vivo conditions in a model of diabetes mellitus, as well as the synergistic effect of both polyphenols in the inhibition of these mediators of cardiovascular damage.

Previous studies showed the effect of HT, DHPG, and their association on neuroprotection and platelet function. Some studies showed a beneficial effect of HT on the alteration of carbohydrate metabolism related to type 2 diabetes mellitus. It was sufficiently demonstrated that both type 1 and type 2 diabetes mellitus increase the risk of a cardiovascular event, in some cases, independently of the complications associated with sustained hyperglycemia.

The main limitation of this study is that it did not delve into the relationship of the two polyphenols studied with the metabolic process of diabetes mellitus. The novelty was the use of a diabetes mellitus model to assess whether this interaction between both polyphenols occurred in diabetes, which is one of the main risk factors for cardiovascular disease. In fact, this model of type 1 diabetes is used as an inducer of cardiovascular damage, rather than as a model for studying compounds that could affect metabolic disease itself. A study that studies the effects of HT and DHPG on the evolution of diabetes is desirable, but perhaps that should be studied in an experimental model of type 2 diabetes, where tissue mechanisms (insulin resistance) are more important. In the type 1 diabetes model used, it is widely recognized that pancreatic insulin production is abolished, and it is postulated as a model that increases all of the profiles (biochemical and morphological) of vascular damage (macro and microangiopathic), which is just what we intended in this study. We are currently developing a study on the effect of these extra-virgin olive oil polyphenols in a type 2 diabetes model, analyzing not only the parameters of vascular damage, but also the metabolic disease itself.

## 5. Conclusions

This study showed that two polyphenols present in EVOO, hydroxytyrosol and 3’,4’-dihydroxyphenylglycol, showed a beneficial effect on various mechanisms that participate in the genesis and evolution of cardiovascular disease in an experimental model of type 1 diabetes mellitus. Likewise, the association of both compounds, in the proportions in which they are found in different types of EVOO, shows a synergistic effect on these mechanisms, mainly in the inhibition of oxidative and nitrosative stress, platelet aggregation, vascular production of prostacyclin, myeloperoxidase, and adhesive proteins (VCAM-1). All of these findings could be important for the development of functional oils enriched in these two polyphenols in the proportion used in this study.

## Figures and Tables

**Table 1:** Body weight, daily chow, and water intake (mean ± standard deviation) of nondiabetic rats (NDR), diabetic control rats (DR), and DR treated with hydroxytyrosol (HT) 5 mg/kg/day p.o. (HT-5), 3′,4′-dihidroxifenilglicol (DHPG) 0.5 or 1 mg/kg/day p.o. (DHPG-0.5, DHPG-1), or their association. *n* = 10 rats per group.

Variable	NDR	DR	HT-5	DHPG-0.5	DHPG-1	HT-5 + DHPG-0.5	HT-5 + DHPG-1
Body weight (g)	460 ± 7.3	347 ± 17.5 ^(+)^	358 ± 20.1	368 ± 17.8	364 ± 15.1	368 ± 23.8	374 ± 16.2
Chow intake (g/day)	19.5 ± 2.0	29.5 ± 3.5 ^(+)^	24.3 ± 3.9	28.2 ± 2.9	23.8 ± 2.5	24.2 ± 2.9	27.2 ± 3.0
Water intake (mL/day)	39.5 ± 5.1	110 ± 6.9 ^(+)^	82.7 ± 13.4	85.0 ± 9.1	82.5 ± 10.5	73.7 ± 10.3	80.7 ± 9.6

^+^*p* < 0.05 with respect to NDR.

**Table 2:** Serum and urine variables (mean ± standard deviation) of nondiabetic rats (NDR), diabetic control rats (DR), and DR treated with hydroxytyrosol (HT) 5 mg/kg/day p.o. (HT-5), 3′,4′-dihidroxifenilglicol (DHPG) 0.5 or 1 mg/kg/day p.o. (DHPG-0.5, DHPG-1), or their association. *n* = 10 rats per group.

Variable	NDR	DR	HT-5	DHPG-0.5	DHPG-1	HT-5 + DHPG-0.5	HT-5 + DHPG-1
Blood glucose (mg/dL)	95.1 ± 5.3	456 ± 10.0 ^(+)^	460 ± 9.7	384 ± 32.5	424 ± 36.7	329 ± 39.8	420 ± 10.8
Total cholesterol (mg/dL)	57.5 ± 7.4	78.7 ± 4.7	68.3 ± 2.5	75.2 ± 7.5	71.0 ± 9.1	70.7 ± 6.7	72.5 ± 4.7
LDL cholesterol (mg/dL)	23.2 ± 5.4	33.5 ± 5.8	30.1 ± 7.8	34.0 ± 6.8	29.2 ± 6.1	28.2 ± 7.1	27.7 ± 4.1
HDL cholesterol (mg/dL)	18.5 ± 2.4	18.4 ± 4.8	28.0 ± 8.1 ^(*)^	25.5 ±5.4	27.5 ± 4.4	24.5 ± 4.1	25.2 ± 2.8
Triglycerides (mg/dL)	67.5± 7.7	105 ± 13.7 ^(+)^	102 ± 6.6	108 ± 6.5	109 ± 13.5	85.5 ± 7.8 ^(*,a)^	110 ± 7.4 ^(*)^

^+^*p* < 0.05 with respect to NDR; * *p* < 0.05 with respect to DR; ^a^ *p* < 0.05 with respect to HT-5, DHPG-0.5 and DHPG-1.

**Table 3:** Variables (mean ± standard deviation) of oxidative and nitrosative stress of nondiabetic rats (NDR), diabetic control rats (DR), and DR treated with hydroxytyrosol (HT) 5 mg/kg/day p.o. (HT-5), 3′,4′-dihidroxifenilglicol (DHPG) 0.5 or 1 mg/kg/day p.o. (DHPG-0.5, DHPG-1), or their association. *n* = 10 rats per group.

Variable	NDR	DR	HT-5	DHPG-0.5	DHPG-1	HT-5 + DHPG-0.5	HT-5 + DHPG-1
TBARS (nmol/mL)	4.0 ± 0.8	8.9 ± 0.7 ^(+)^	4.7 ± 0.4 ^(*,c)^	6.7 ± 0.4 ^(*)^	6.5 ± 0.5 ^(*)^	5.1 ± 0.6 ^(*)^	2.4 ± 0.4 ^(*,c)^
oxLDL (ng/mL)	14.0 ± 0.9	24.7 ± 1.5 ^(+)^	17.3 ± 0.5 ^(*)^	16.7 ± 2.3 ^(*)^	17.8 ± 2.1 ^(*)^	12.3 ± 1.3 ^(*,b)^	12.0 ± 0,6 ^(*,b)^
8-OHdG (ng/mL)	15.6 ± 0.6	27.5 ± 1.5 ^(+)^	15.5 ± 1.4 ^(*)^	16.5 ± 1.1 ^(*)^	14.9 ± 1.6 ^(*)^	12.2 ± 2.1 ^(*,a)^	15.4 ± 0.7 ^(*,a)^
8-isoprostanes (ng/mg urine creatinine)	6.5 ± 0.5	9.2 ± 0.6 ^(+)^	5.6 ± 0.6 ^(*)^	4.8 ± 0.7 ^(*)^	4.8 ± 0.8 ^(*,e)^	4.2 ± 0.5 ^(*)^	2.8 ± 0.3 ^(*,e)^
GHS (nmol/mL)	131 ± 5.4	90.4 ± 7.2 ^(+)^	114 ± 4.8 ^(*)^	103 ± 3.5 ^(*)^	111 ± 6.8 ^(*)^	140 ± 5.3 ^(*,f)^	132 ± 4.5 ^(*,f)^
GSHpx (nmol/min/mL)	7.0 ± 0.8	18.7 ± 1.6 ^(+)^	10.7 ± 1.0 ^(*)^	13.3 ± 1.5 ^(*)^	15.6 ± 0.2 ^(*,e)^	15.7 ± 0.5 ^(*,e)^	15.6 ± 0.9 ^(*,e)^
TAC (U/mL)	19.8 ± 0.7	12.1 ± 0.8 ^(+)^	17.1 ± 0.6 ^(*,b)^	13.1 ± 0.6	15.2 ± 0.8 ^(*)^	16.6 ± 0.5 ^(*)^	17.7 ± 0.4 ^(*,b)^
3-nitrotyrosine (pg/mL)	1.9 ± 0.2	6.7 ± 0.4 ^(+)^	3.5 ± 0.3 ^(*,d)^	5.4 ± 0.08	4.6 ± 0.1 ^(*)^	2.6 ± 0.3 ^(*,b)^	1.9 ± 0.09 ^(*,c)^

8-OHdG: 8-hydroxy-2-deoxyguanosine; GSH: reduced glutathione; GSHpx: glutathione peroxidase activity; oxLDL: oxidized low-density lipoprotein; TAC: total antioxidant capacity; TBARS: thiobarbituric acid-reactive substances. ^+^ *p* < 0.05 with respect to NDR; * *p* < 0.05 with respect to DR. ^a^ *p* < 0.05 with respect to DHPG-0.5 ^b^ *p* < 0.05 with respect to DHPG-0.5 and DHPG-1; ^c^ *p* < 0.05 with respect to DHPG-0.5, DHPG-1 and HT-5+DHPG-0.5; ^d^ *p* < 0.05 with respect to DHPG both alone and associated with HT; ^e^ *p* < 0.05 with respect to HT and DHPG-0.5; ^f^ *p* < 0.05 with respect to HT, DHPG-0.5 and DHPG-1.

**Table 4:** Cardiovascular variables (mean ± standard deviation) of oxidative and nitrosative stress of nondiabetic rats (NDR), diabetic control rats (DR), and DR treated with hydroxytyrosol (HT) 5 mg/kg/day p.o. (HT-5), 3′,4′-dihidroxifenilglicol (DHPG) 0.5 or 1 mg/kg/day p.o. (DHPG-0.5, DHPG-1), or their association. *n* = 10 rats per group.

Variable	NDR	DR	HT-5	DHPG-0.5	DHPG-1	HT-5 + DHPG-0.5	HT-5 + DHPG-1
Imax (ohms)	12.8 ± 2.1	23.1 ± 2.1 ^(+)^	8.8 ± 1.4 ^(*)^	10.7 ± 1.3 ^(*)^	6.9 ± 1.5 ^(*)^	3.0 ± 0.7 ^(*,a)^	3.2 ± 0.7 ^(*,a)^
11-dH-TxB_2_ (ng/mg urine creatinine)	3.9 ± 0.9	9.7 ± 1.1 ^(+)^	4.4 ± 1.2 ^(*)^	8.9 ± 1.8	6.8 ± 0.9	5.6 ± 1.3	7.8 ± 1.6
6-keto-PGF_1__α_ (pg/mg urine creatinine)	14.0 ± 1.4	6.8 ± 1.0 ^(+)^	10.8 ± 1.6 ^(*)^	11.1 ± 0.9 ^(*)^	13.1 ± 1.9 ^(*)^	16.6 ± 1.5 ^(*,c)^	18.7 ± 2.8 ^(*,c)^
MPOx (ng/mL)	0.8 ± 0.08	2.5 ± 0.3 ^(+)^	0.7 ± 0.2 ^(*)^	0.8 ± 0.1	0.8 ± 0.09	0.5 ± 0.1 ^(*)^	0.4 ± 0.08 ^(*)^
VCAM-1 (ng/mL)	4.8 ± 0.8	9.7 ± 0.8 ^(+)^	4.1 ± 0.4 ^(*)^	7.9 ± 0.6 ^(*)^	6.1 ± 0.8 ^(*)^	4.6 ± 0.5 ^(*)^	3.4 ± 0.8 ^(*,b)^

Imax: maximum intensity of platelet aggregation induced with collagen. 6-keto-PGF_1α_: 6-keto-prostaglandin F_1α_; 11-dH-TxB_2_: 11-dehydro-tromboxane B_2_. MPOx: myeloperoxidase. * *p* < 0.05 with respect to DR. ^a^ *p* < 0.05 with respect to DHPG-0.5; ^b^ *p* < 0.05 with respect to DHPG-0.5, DHPG-1 and HT-5 + DHPG-0.5; ^c^ *p* < 0.05 with respect to HT, DHPG-0.5 and DHPG-1.

**Table 5:** Morphometric variables from aortic sections (mean ± standard deviation) of nondiabetic rats (NDR), diabetic control rats (DR), and DR treated with hydroxytyrosol (HT) 5 mg/kg/day p.o. (HT-5), 3′,4′-dihidroxifenilglicol (DHPG) 0.5 or 1 mg/kg/day p.o. (DHPG-0.5, DHPG-1), or their association. *n* = 10 rats per group.

Variable	NDR	DR	HT-5	DHPG-0.5	DHPG-1	HT-5 + DHPG-0.5	HT-5 + DHPG-1
Aortic thickness (µm)	108 ± 5.8	144 ± 5.2 ^(+)^	113 ± 7.9 ^(*)^	120 ± 3.3 ^(*)^	190 ± 9.4	117 ± 4.2 ^(*)^	118 ± 4.8 ^(*)^
Smooth muscle cells (n/10^5^ µm^2^)	41.9 ± 2.2	55.2 ± 2.8 ^(+)^	44.5 ± 3.5 ^(*)^	41.2 ± 2.1 ^(*)^	41.3 ± 1.9 ^(*)^	38.1 ± 3.8 ^(*)^	44.0 ± 1.9 ^(*)^

^+^*p* < 0.05 with respect to NDR; * *p* < 0.05 with respect to DR.

## Data Availability

The data presented in this study are available in article.
